# Supplementary material for: Tead1a Initiates Transcriptional Priming Through the TEAD1a/YAP‐Notch1‐Spi1/Cebpα Axis to Promote Neutrophil Fate
Source: Adv Sci (Weinh). 2025 Aug 28;12(41):e05441. doi: 10.1002/advs.202505441 (PMC12591127; doi:10.1002/advs.202505441)
Supplement: Supplementary file 4 — Supplementary Dataset [file ADVS-12-e05441-s004.zip › 3-4.zip!/3-4/02-4.cluster/cluster11/Enrich/ego_all.pdf]

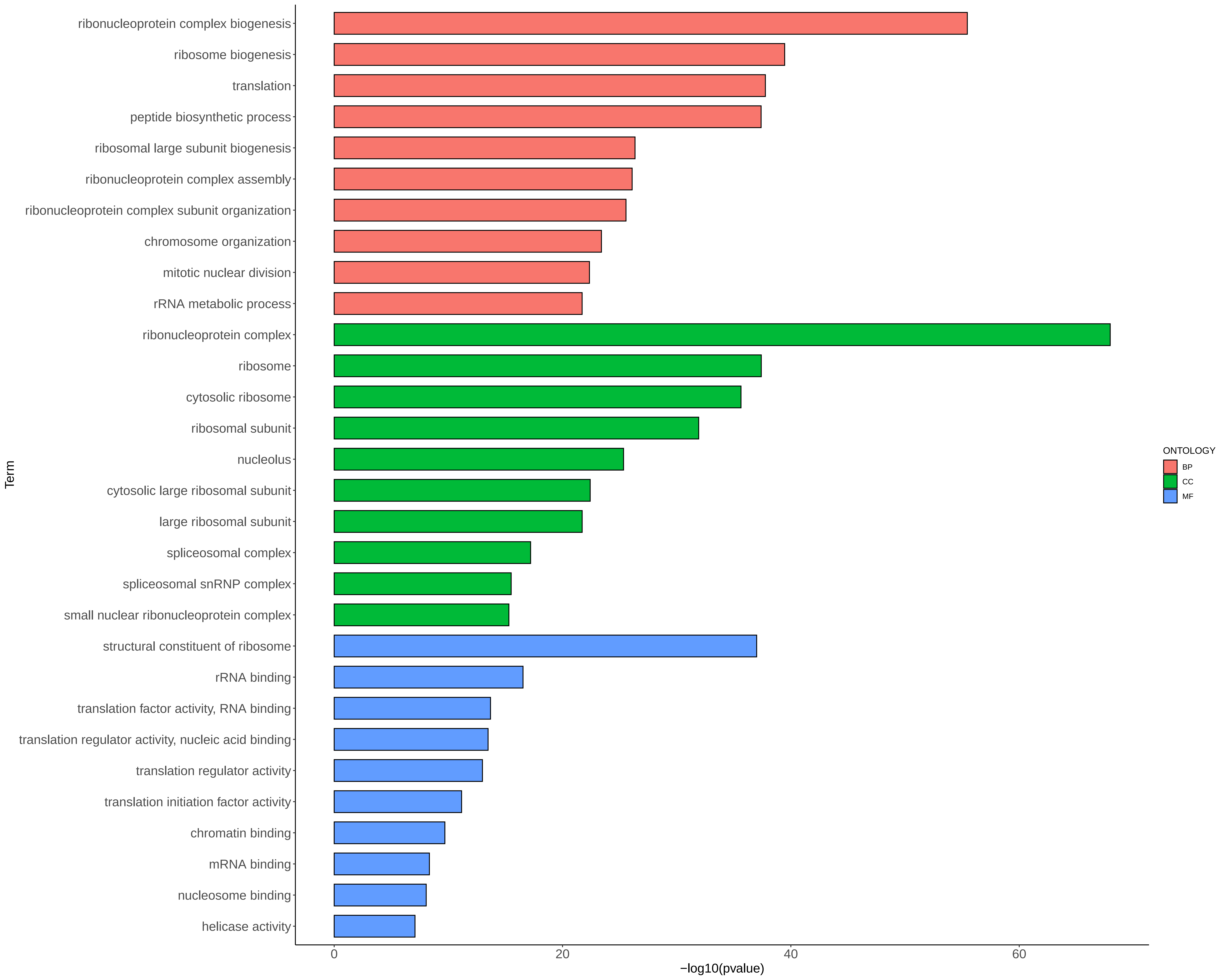

ribonucleoprotein complex biogenesis
ribosome biogenesis
translation
peptide biosynthetic process
ribosomal large subunit biogenesis
ribonucleoprotein complex assembly
ribonucleoprotein complex subunit organization
chromosome organization
mitotic nuclear division
rRNA metabolic process
ribonucleoprotein complex
ribosome
cytosolic ribosome
ribosomal subunit
nucleolus
cytosolic large ribosomal subunit
large ribosomal subunit
spliceosomal complex
spliceosomal snRNP complex
small nuclear ribonucleoprotein complex
structural constituent of ribosome
rRNA binding
translation factor activity, RNA binding
translation regulator activity, nucleic acid binding
translation regulator activity
translation initiation factor activity
chromatin binding
mRNA binding
nucleosome binding
helicase activity
Term
0
20
40
60
−log10(pvalue)
ONTOLOGY
BP
CC
MF
